# Factors associated with workarounds in barcode‐assisted medication administration in hospitals

**DOI:** 10.1111/jocn.15217

**Published:** 2020-02-28

**Authors:** Willem van der Veen, Katja Taxis, Hans Wouters, Hester Vermeulen, David W. Bates, Patricia M. L. A. van den Bemt, the BCMA Study Group, Michiel Duyvendak, Karen Oude Luttikhuis, Johannes J. W. Ros, Erwin C. Vasbinder, Maryam Atrafi, Bjorn Brasse, Iris Mangelaars

**Affiliations:** ^1^ Unit PharmacoTherapy, Epidemiology & Economics Faculty of Science and Engineering University of Groningen Groningen The Netherlands; ^2^ General Practitioners Research Institute University of Groningen Groningen The Netherlands; ^3^ Department of IQ Healthcare Scientific Center for Quality of Healthcare Radboud Institute of Health Sciences Nijmegen The Netherlands; ^4^ Harvard Medical School and Brigham and Woman's Hospital Boston MA USA; ^5^ Department Clinical Pharmacy and Pharmacology University Medical Center Groningen Groningen The Netherlands

**Keywords:** Barcode‐assisted Medication Administration, health information technology, medication safety, nurse‐performed‐workarounds, nurse workload, quality of care

## Abstract

**Aims and objectives:**

To identify that workarounds (defined as “informal temporary practices for handling exceptions to normal procedures or workflow”) by nurses using information technology potentially compromise medication safety. Therefore, we aimed to identify potential risk factors associated with workarounds performed by nurses in Barcode‐assisted Medication Administration in hospitals.

**Background:**

Medication errors occur during the prescribing, distribution and administration of medication. Errors could harm patients and be a tragedy for both nurses and medical doctors involved. Interventions to prevent errors have been developed, including those based on information technology. To cope with shortcomings in information technology‐based interventions as Barcode‐assisted Medication Administration, nurses perform workarounds. Identification of workarounds in information technology is essential to implement better‐designed software and processes which fit the nurse workflow.

**Design:**

We used the data from our previous prospective observational study, performed in four general hospitals in the Netherlands using Barcode techniques, to administer medication to inpatients.

**Methods:**

Data were collected from 2014–2016. The disguised observation was used to gather information on potential risk factors and workarounds. The outcome was a medication administration with one or more workarounds. Logistic mixed models were used to determine the association between potential risk factors and workarounds. The STROBE checklist was used for reporting our data.

**Results:**

We included 5,793 medication administrations among 1,230 patients given by 272 nurses. In 3,633 (62.7%) of the administrations, one or more workarounds were observed. In the multivariate analysis, factors significantly associated with workarounds were the medication round at 02 p.m.–06 p.m. (adjusted odds ratio [OR]: 1.60, 95% CI: 1.05–2.45) and 06 p.m.–10 p.m. (adjusted OR: 3.60, 95% CI: 2.11–6.14) versus the morning shift 06 a.m.–10 a.m., the workdays Monday (adjusted OR: 2.59, 95% CI: 1.51–4.44), Wednesday (adjusted OR: 1.92, 95% CI: 1.2–3.07) and Saturday (adjusted OR: 2.24, 95% CI: 1.31–3.84) versus Sunday, the route of medication, nonoral (adjusted OR: 1.28, 95% CI: 1.05–1.57) versus the oral route of drug administration, the Anatomic Therapeutic Chemical classification‐coded medication “other” (consisting of the irregularly used Anatomic Therapeutic Chemical classes [D, G, H, L, P, V, Y, Z]) (adjusted OR: 1.49, 95% CI: 1.05–2.11) versus Anatomic Therapeutic Chemical class A (alimentary tract and metabolism), and the patient–nurse ratio ≥6–1 (adjusted OR: 5.61, 95% CI: 2.9–10.83) versus ≤5–1.

**Conclusions:**

We identified several potential risk factors associated with workarounds performed by nurses that could be used to target future improvement efforts in Barcode‐assisted Medication Administration.

**Relevance to clinical practice:**

Nurses administering medication in hospitals using Barcode‐assisted Medication Administration frequently perform workarounds, which may compromise medication safety. In particular, nurse workload and the patient–nurse ratio could be the focus for improvement measures as these are the most clearly modifiable factors identified in this study.

**What does this paper contribute to the wider global clinical community**

- Nurses administering medication using Barcode‐assisted Medication Administration frequently perform workarounds.
- Potential risk factor associated with these workarounds was the nurse workload.
- Especially, the nurse workload found in infrequently performed nursing procedures and the patient–nurse ratio could be the focus for improvement measures in medication administration using Barcode‐assisted Medication Administration.

## 1 INTRODUCTION

Nurses are in the front‐line caring for hospital inpatients, which involves a multitude of complex tasks, including the administration of prescribed medication to patients. Nurses perform the last step in the multifaceted process performed by several health professionals of prescribing and administering medication, capable of protecting patients from medication errors. Therefore, they have a crucial role in medication safety.

Electronic Barcode‐assisted Medication Administration (BCMA) systems have been developed to help nurses to reduce medication administration errors (Hutton, Ding, & Wellman, 2017; Poon et al., 2010). The BCMA system checks the information obtained by scanning both the barcode on the medication package and the bar‐coded patient wristband against the information provided by the hospital's electronic prescribing system. An electronic alert is given in case of a mismatch. In some BCMA systems, nurses also have a personal barcode so that the individuals administering the medication are registered automatically. Several studies have shown a substantial decrease in medication administration errors following the implementation of BCMA systems in hospitals (Berdot et al., 2016; Hassink, Essenberg, Roukema, & van den Bemt, 2013; Helmons, Wargel, & Daniels, 2009; Maaskant et al., 2015; Poon et al., 2010).

However, Information Technolgy (IT) based interventions as BCMA are not always used as instructed and required, and nurses perform workarounds. Workarounds (defined as “informal temporary practices for handling exceptions to normal procedures or workflow”) by nurses using IT potentially compromise medication safety (van der Veen, van den Bemt, Wouters, et al., 2017). Until now, the factors that force nurses to carry out workaround were unclear.

### 1.1 Aim

This study aimed to identify potential risk factors associated with nurse workarounds in BCMA in order to explore why nurses perform workarounds.

## 2 BACKGROUND

Medication errors occur during the prescribing, distribution and administration of medication (Krahenbuhl‐Melcher et al., 2007; Lisby, Nielsen, & Mainz, 2005). Errors may not only harm patients but could also be a tragedy for healthcare workers as nurses. Several interventions to prevent errors have been developed, including those based on information technology (IT). IT‐based interventions are most promising because they have the potential to structure and standardise processes as prescribing and administration of medication (Bates, 2000; Kaushal & Bates, 2002; Seidling & Bates, 2016). In practice, IT‐based interventions in health care, such as BCMA, are not always used as instructed and required or fit the daily workflow (Cheung et al., 2014; van der Veen, van den Bemt, Wouters, et al., 2017). Nurses adopt so‐called workarounds (Ash, Berg, & Coiera, 2004; Koppel, Wetterneck, Telles, & Karsh, 2008a; Rack, Dudjak, & Wolf, 2012) to cope with IT‐based interventions as BCMA in daily practice. Workarounds were defined as “informal temporary practices for handling exceptions to normal workflow” (Kobayashi, Fussell, Xiao, & Seagull, 2005). While performing workarounds, the nurse workflow can be changed, either once, temporarily or even over an extended period, but identity, purpose or construction of the system remains unchanged (Alter, 2014). End users of BCMA such as the nursing staff occasionally practice workarounds to deal with perceived issues, which may relate to lack of confidence in technology, the time that using this technology takes or other issues relating to hardware, programming, screen design, user knowledge or communication problems (Debono et al., 2013; Koppel, 2014; Patterson, 2018). Examples of workarounds performed by nurses using BCMA are as follows: not scanning at all, scanning patient's barcodes remotely (i.e. not the actual wristbands of patients), ignoring signals or alerts or scanning of medication for multiple patients at the same time. Identification of workarounds in IT is essential to implement better‐designed software and processes which fit the nurse workflow. As seen in qualitative research (Cresswell, Mozaffar, Lee, Williams, & Sheikh, 2016), workarounds can improve efficiency, but may also create new risks and compromise the safety and effectiveness of patient care. A lack of coherence between the wishes and expectations of healthcare providers, patients and technological capabilities could be the cause of this. Suggested is that, to avoid this, healthcare workers such as nurses, pharmacists and medical doctors should already be involved in the implementation phase of new IT‐based systems (Blijleven, Koelemeijer, Wetzels, & Jaspers, 2017; Koppel, Smith, Blythe, & Kothari, 2015; van der Veen, de Gier, van der Schaaf, Taxis, & van den Bemt, 2013). In our previous study in four hospitals in the Netherlands, we showed that workarounds are associated with medication administration errors (van der Veen, van den Bemt, Wouters, et al., 2017). Other research identified circumstantial factors for performing workarounds but focused mainly on the usability of the BCMA system (Debono et al., 2013; Holden, Rivera‐Rodriguez, Faye, Scanlon, & Karsh, 2013; Patterson, Rogers, Chapman, & Render, 2006). Our data (van der Veen, van den Bemt, Wouters, et al., 2017) provide the opportunity to study quantitatively (instead of qualitatively) the potential risk factors associated with workarounds in IT‐based intervention BCMA. This may be considered as a first step to develop interventions intended to reduce the frequency of nurse workarounds in the use of BCMA in hospitals.

## 3 DESIGN

We used the data (van der Veen, van den Bemt, Wouters, et al., 2017) of our multicentre prospective observational study conducted in adult patients (aged 18 years and older) admitted to four hospitals in the Netherlands using BCMA in the medication administration process (at the time of planning our study, eight hospitals in the Netherlands were using BCMA‐based systems). The research project was started in 2014, and enrolment was completed at the end of 2016. Both a detailed version of the study protocol (van der Veen, van den Bemt, Bijlsma, de Gier, & Taxis, 2017) and the analysis of the association between workarounds and medication errors (van der Veen, van den Bemt, Wouters, et al., 2017) have been published before. The study was registered in the “Dutch trial register” with trial ID NTR4355. Data were anonymised following Dutch privacy legislation (van der Veen, van den Bemt, Wouters, et al., 2017).

### 3.1 Ethics

The regional medical ethics committee (in Dutch: “Regionale Toetsingscommissie Patientgebonden Onderzoek RTPO”) approved the study protocol on 22 May 2014.

## 4 METHODS

The STROBE checklist for reporting cohort, case–control and cross‐sectional studies was used (File S1).

### 4.1 Setting

All four included hospitals operated electronic prescribing of medication and BCMA, each using different software for both the prescribing and the BCMA part. After scanning the barcode of both the patient and the medication, the BCMA system checked whether the patient and the medication matched the physician's prescription.

To facilitate the scanning of medication, pharmacy technicians dispensed unit‐dose barcode‐labelled medication. Medication rounds were scheduled on the following times: 6 a.m.–10 a.m., 10 a.m.–2 p.m., 6 p.m.–8 p.m. and 8 p.m.–10 p.m. Per the medication round, one nurse was responsible for medication administration. Nurse trainees were supervised by registered nurses (van der Veen, van den Bemt, Wouters, et al., 2017).

### 4.2 Participants

Patients on participating nursing wards of four Dutch hospitals using BCMA to administer medication were included; patients aged 17 years and younger were excluded (van der Veen, van den Bemt, Bijlsma, et al., 2017). These were four out of eight hospitals using BCMA in the Netherlands at the time of planning the study. We included wards from the following areas: cardiology, pulmonary diseases, geriatrics, internal medicine, neurology, surgery and orthopaedics. The observers (three undergraduate students from the schools of pharmacy of the universities of Groningen and Utrecht in the Netherlands, who were all well trained in the technique of observation and who all had to pass an examination in order to be appointed as an observer) accompanied nurses working on the selected wards during the selected drug rounds. The observed nurses were aware of the fact that they were being observed, but not about the precise nature of the data, which were collected (disguised observation).

All the nurses agreed to be observed. Based on Dutch regulation, nurses in training were not responsible for nursing actions but worked under the supervision of a registered nurse who has the final responsibility for the actions of the nurse in training. For this reason, we did not distinguish between a registered nurse and a training nurse in the final analysis.

### 4.3 Data collection

The disguised observation method (Dean & Barber, 1999, 2001; Smeulers, Hoekstra, van Dijk, Overkamp, & Vermeulen, 2013) was used to collect data on potential risk factors and workarounds (van der Veen, van den Bemt, Bijlsma, et al., 2017). In practice, each observer accompanied the nurse who administered the medication using BCMA and observed the administration of each medication to the patient. The following observation schedule was followed: at least three rounds were observed each day of the week, with a weekly minimum of 21 medication administration rounds. Details of the drug administration to the patient were documented using a case report form (van der Veen, van den Bemt, Bijlsma, et al., 2017). In case the observer noticed a potentially dangerous error, he or she intervened for ethical reasons, while retaining these observations in the data set. Incomplete observations were excluded.

### 4.4 Definition and classification

We defined workarounds using the definition of Kobayashi as “informal temporary practices for handling exceptions to the normal workflow” (Kobayashi et al., 2005). Workarounds were classified as described earlier (van der Veen, van den Bemt, Bijlsma, et al., 2017) using a self‐developed classification system and observation form based on the research of Koppel (Koppel et al., 2008). Workarounds were classified as procedural‐based, related to patient identification, the scanning process, the computers or scanner alert signals, and other workflow procedures, or nurse‐work‐related. To determine whether a workaround took place, the observers compared their observation records after each medication administration round to the hospital or ward procedures and local guidelines on the BCMA process (van der Veen, van den Bemt, Bijlsma, et al., 2017).

### 4.5 Outcome measure and potential risk factors

The proportion of medication administrations with one or more workarounds was the main outcome measure. Potential risk factors associated with workarounds were selected based on the research of Van den Bemt (van den Bemt, 2006; van den Bemt, Idzinga, Robertz, Kormelink, & Pels, 2009), Schimmel (Schimmel, Becker, van den Bout, Taxis, & van den Bemt, 2011), Driscoll (Driscoll et al., 2018), Aiken (Aiken et al., 2014), Spetz (Spetz, Donaldson, Aydin, & Brown, 2008), Donaldson and Shapiro (Donaldson & Shapiro, 2010) and Wise (Wise, 2016). The following factors were included to analyse their association with workarounds: general characteristics (ward type, time of medication round, day of the week, patient age and gender), medication characteristics (percentage barcoded medication, route of administration, i.e. oral vs. nonoral), the first level of the Anatomic Therapeutic Chemical (ATC) classification code system (Anonymous, 2012, 2017) (the ATC code system is an international drug classification scheme, aimed to categorise the active ingredients of drugs according to the organ or system on which they act and their therapeutic, pharmacological and chemical properties, developed by the World Health Organization [WHO] (Table 2]), nurse characteristics (work experience [≤24 months, >24 months]), nurse workload characteristics during the medication shift (i.e. the number of medications per patient [1, 2, ≥3]) and the patient–nurse ratio (i.e. the number of beds occupied by patients divided by the number of registered nurses on that ward during one shift).

### 4.6 Statistical analysis

The association between potential risk factors and workarounds was analysed using logistic mixed models using a similar statistical approach as in our previous study (van der Veen, van den Bemt, Wouters, et al., 2017). To take into account the potential dependency of observations (i.e. more than one observation was made for each nurse), a random intercept at the ward and the nurse level was included in the models. Owing to observed multicollinearity between the training of the nurse (student nurse vs. registered nurse) and the work experience (≤24 months vs. >24 months) of the nurse, we only included work experience as a variable in the model. The type of hospital (general vs. training hospital) corresponded with the percentage of medication supplied with a barcode (<99% vs. ≥99%). Therefore, we did not include the hospital type in the analysis. First, univariate analyses were performed in which we examined the factors individually. Subsequently, multivariate analysis was performed, including the percentage of barcoded medication, type of nursing department, the day of the week, time of the medication round, ATC classes, the number of drugs per round, route of administration and the patient–nurse ratio as the independent variables. Mixed model analyses were carried out using mlwin version 6.3. All other analyses were carried out using spss version 23.0, similar to our protocol and our previous study (van der Veen, van den Bemt, Bijlsma, et al., 2017; van der Veen, van den Bemt, Wouters, et al., 2017).

## 5 RESULTS

We observed 6,021 medication administrations in patients admitted to four hospitals, of which 228 (3.8%) were excluded because they were incomplete. The included 5,793 medication administrations were given to 1,230 inpatients by 272 nurses. In 3,633, medication administrations (62.7%), one or more workarounds, were observed (van der Veen, van den Bemt, Wouters, et al., 2017). The characteristics of the study hospitals and the nurses are presented in Table 1.

**Table 1:** Characteristics of study hospitals (*N* = 4) and nurses (*N* = 272)

Characteristics	Category	*N*	%
Hospitals (*n* = 4)			
Location	Rural area	2	50
	Urban area	2	50
Number of beds	200–400	1	25
	401–600	2	50
	601–800	1	25
Hospital type	General	3	75
	Teaching	1	25
Hospital BCMA experience	2–4 years	1	25
	4–6 years	2	50
	6–8 years	1	25
Nurses (*n* = 272)			
Gender	Male	24	8.8
	Female	248	91.2
Education level	Student nurse	33	12.1
	Registered nurse	236	86.7
	Unknown^a^	3	1.2
Experience as a registered nurse	≤24 months	36	15.3
	>24 months	198	83.9
	Unknown	2	0.8
Registered nurse BCMA experience	≤6 months	28	11.9
	>6 months	206	87.3
	Unknown	2	0.8
Nursing ward	Cardiology	39	14.3
	Pulmonary diseases	29	10.7
	Geriatrics	15	5.5
	Internal medicine	53	19.5
	Neurological diseases	35	12.9
	Surgical diseases	60	22.1
	Orthopaedics	30	11.0
	Other type of nursing ward	11	4.0
Mean patient–nurse ratio on wards during medication administration shifts	Hospital 1	5	
	Hospital 2	4	
	Hospital 3	7	
	Hospital 4	6	

^a^ These 3 nurses were responsible for 48 medication administrations, 25 without a workaround and 23 with a workaround.

Procedural workarounds (as not scanning at all) were most common (*n* = 1,307, 36%). Other workarounds observed were patient scanning‐related (as no barcode wristband on the patient; *n* = 1,017, 28%) and medication scanning‐related (including scanning before actual administration of medication, scanning medication for more than one patient at a time, and ignoring computer or scanner alerts; *n* = 400, 11%). The observers did not have to intervene during the observation period.

In the multivariate analysis, factors significantly associated with workarounds were the medication round at 02 p.m.–06 p.m. (adjusted OR: 1.60, 95% CI: 1.05–2.45) and 06 p.m.–10 p.m. (adjusted OR: 3.60, 95% CI: 2.11–6.14) versus the morning shift 06 a.m.–10 a.m., the workdays Monday (adjusted OR: 2.59, 95% CI: 1.51–4.44), Wednesday (adjusted OR: 1.92, 95% CI: 1.2–3.07), Saturday (adjusted OR: 2.24, 95% CI: 1.31–3.84) versus Sunday, the route of medication, nonoral (adjusted OR: 1.28, 95% CI: 1.05–1.57) versus the oral route of drug administration, the Anatomic Therapeutic Chemical (ATC) classification‐coded (Table 2) medication “other” (consisting of infrequently used ATC classes [D, G, H, L, P, V, Y, Z]) (adjusted OR: 1.49, 95% CI: 1.05–2.11) versus ATC class A (alimentary tract and metabolism) and the patient–nurse ratio, ≥6–1 (adjusted OR: 5.61, 95% CI: 2.90–10.83) versus ≤5–1 (Table 3). Factors not significantly associated with workarounds were the ward type, the patient age and gender, the percentage of barcoded medication, the number of medications per patient and the nurse work experience. Observers did not record the level of education of three nurses. They were responsible for 48 administrations (23 with a workaround and 25 of them without a workaround). Those figures did not significantly change the workaround percentage (62.8% workarounds instead of 62.7%).

**Table 2:** Anatomic Therapeutic Chemical (ATC) classification system

ATC Code	Drugs related to organ system or use
A	Alimentary tract and metabolism
B	Blood and blood‐forming organs
C	Cardiovascular system
D	Dermatological medication
G	Genitourinary system and sex hormones
H	Systemic hormonal preparations, excluding sex hormones and insulins
J	Anti‐infective for systemic use
L	Antineoplastic and immunomodulating agents
M	Muscular–skeletal system
N	Nervous system
P	Antiparasitic products, insecticides and repellents
R	Respiratory system
S	Sensory organs, eye, nose, ear
V	Various drugs
Y	Not supplied
Z	Not relevant

**Table 3:** Unadjusted and adjusted associations between factors and workarounds in barcode‐assisted medication administration

Factors	Factor	No WA (*N*)	%	WA (*N*)	%	Unadjusted OR^b^	95% CI	Adjusted OR^c^	95% CI
General factors									
Ward type	Cardiology	341	5.89	682	11.77	Ref.^a^	—	Ref.^a^	—
	Pulmonary diseases	380	6.56	278	4.80	0.30	0.08–1.13	0.29	0.07–1.29
	Geriatrics	159	2.74	122	1.93	0.34	0.07–1.58	0.82	0.14–4.84
	Internal medicine	406	7.01	611	10.55	0.83	0.30–2.31	1.31	0.40–4.25
	Neurological diseases	219	3.78	425	7.34	0.96	0.28–3.30	0.91	0.23–3.60
	Surgical diseases	406	7.01	1,008	17.40	0.90	0.32–2.51	1.45	0.45–4.70
	Orthopaedics	153	2.64	447	7.72	1.08	0.29–4.06	0.77	0.18–3.33
	Other type of nursing ward, for example day care	96	1.66	60	1.04	0.39	0.05–2.95	0.90	0.09–8.7
Time of medication run	06 a.m.–10 a.m.	1509	26.05	1775	30.64	Ref.^a^	—	Ref.^a^	—
	10 a.m.–02 p.m.	98	1.69	160	2.76	1.57	1.01–2.42	1.57	0.93–2.66
	02 p.m.–06 p.m.	472	8.15	472	8.15	1.57	1.11–2.22	1.60	1.05–2.45
	06 p.m.–10 p.m.	81	1.40	1,226	21.16	8.79	6.39–12.1	3.60	2.11–6.14
Day of the week	Sunday	159	2.74	374	6.46	Ref.^a^	—	Ref.^a^	—
	Monday	228	3.94	504	8.07	1.91	1.24–2.96	2.59	1.51–4.44
	Tuesday	360	6.21	572	9.87	1.43	0.94–2.17	1.34	0.79–2.26
	Wednesday	377	6.51	681	11.76	1.19	0.80–1.75	1.92	1.20–3.07
	Thursday	405	6.99	723	12.48	1.57	1.02–2.39	1.58	0.95–2.62
	Friday	290	5.01	331	5.71	1.20	0.75–1.91	1.66	0.92–3.00
	Saturday	305	5.26	374	6.46	1.57	1.02–2.42	2.24	1.31–3.84
Patient age^d^	<74 years of age	1,072	18.51	1855	32.02	Ref.^a^	—	Ref.^a^	—
	≥74 years of age	1,088	18.78	1778	30.69	1.02	0.90–1.15	0.95	0.82–1.1
Patient gender	Women	1,037	17.90	1657	28.60	Ref.^a^	—	Ref.^a^	—
	Men	1,123	19.39	1976	34.11	0.89	0.79–1.01	0.89	0.78–1.03
Medication factors									
% barcoded medication	≥99%	713	12.31	815	14.07	Ref.^a^	—	Ref.^a^	—
	<99%	1,447	24.98	2,818	48.64	0.65	0.28–1.53	1.22	0.47–3.15
Route of administration	Oral medication	1831	31.61	2,951	50.94	Ref.^a^	—	Ref.^a^	—
	Nonoral route^e^	329	5.68	682	11.77	1.28	1.10–1.49	1.28	1.05–1.57
ATC^f^ code (first level)	ATC A	556	9.60	757	13.07	Ref.^a^	—	Ref.^a^	—
	ATC B	182	3.14	381	6.58	1.14	0.92–1.42	0.99	0.76–1.28
	ATC C	479	8.27	620	10.70	0.96	0.81–1.13	0.96	0.79–1.15
	ATC J	67	1.16	187	3.23	1.59	1.17–2.16	1.42	0.99–2.04
	ATC M	68	1.17	104	1.80	1.13	0.80–1.59	1.12	0.77–1.63
	ATC N	530	9.15	1,095	18.90	1.16	0.99–1.36	1.01	0.84–1.21
	ATC R	119	2.05	235	4.06	1.17	0.90–1.51	0.89	0.64–1.23
	ATC S	86	1.48	110	1.90	0.98	0.71–1.35	0.85	0.58–1.24
	Infrequently used classes (D, G, H, L, P, V, Y, Z)^g^	73	1.26	144	2.49	1.48	1.07–2.04	1.49	1.05–2.11
Nurse factors									
Work experience	<24 months	355 hr	6.18	541^i^	9.42	Ref.^a^	—	Ref.^a^	—
	≥24 months	1,780 hr	30.98	3,069^i^	53.42	1.30	0.86–1.96	1.03	0.6–1.76
Workload factors									
Drugs per round per patient	1	52	0.90	187	3.23	Ref.^a^	—	Ref.^a^	—
	2	130	2.24	367	6.34	0.92	0.65–1.30	0.97	0.63–1.50
	≥3	1978	34.14	3,079	53.15	0.81	0.60–1.10	0.96	0.66–1.41
Patient–nurse ratio	≤5 to 1	1755	30.30	1,412	24.37	Ref.^a^	—	Ref.^a^	—
	≥6 to 1	405	6.99	2,221	38.34	12.21	8.14–18.31	5.61	2.90–10.83

^a^ Reference category.

^b^ Odds ratio.

^c^ Adjusted for all variables shown in table.

^d^ Age of 74 is median age.

^e^ Numbers nonoral routes; inhalation 414, parenteral 240, sublingual 118, eye drops 69, dermal drugs 56, other route 114.

^f^ Anatomic Therapeutic Chemical (ATC) classification.

^g^ Other, infrequently used ATC classes D, G, H, L, P, V, Y, Z (in which we observed a total of 217 administrations, range 2–75).

^h^ 25 missing values from 3 nurses.

^i^ 23 missing values from 3 nurses.

## 6 DISCUSSION

Potential risk factors associated with workarounds were the day of the week, the timing of the medication administration, the route of administration, the administration of medication from irregularly used ATC classes and the patient–nurse ratio. Other factors, such as the percentage of barcoded medication and work experience, were not associated with workarounds. These results can be used to help target efforts to reduce the frequency of workarounds in the future.

Procedures should be reviewed critically to ensure that nonorally administered medication can be administered correctly using the BCMA system. Furthermore, nurses need to be well trained to perform infrequent nursing procedures. The association of the nonoral route of administration with workarounds may have several causes. For example, the dermal application, as well as inhalation, is often left to the patient self‐administering this medication. This may enhance the risk of workarounds because nurses may forget to scan such medication. Another example is a parenteral medication that needs handling to make it ready to administer: the original vial with infusion powder may contain a barcode, but the infusion bag with the added drug may not be barcoded. The handling of infrequently used medication (as expressed by the ATC class “other”) may lead to workarounds because of the nurses not being familiar with administering this medication.

A higher patient–nurse ratio was also associated with workarounds. This finding is in line with other studies finding associations between the number of nursing staff and quality of care for hospitalised patients (Ball, Murrells, Rafferty, Morrow, & Griffiths, 2014; Driscoll et al., 2018; Goedhart, van Oostveen, & Vermeulen, 2017; Wise, 2016). Death rates in British hospitals with nurses caring for six or fewer patients were 20% lower than in hospitals with nurses caring for ten or more patients (Griffiths, Ball, Murrells, Jones, & Rafferty, 2016). Little is known about the optimal patient–nurse ratio, and ratios may vary by time of day and patient awareness. In California, the USA, rules require a patient–nurse ratio of one nurse to every five patients (Donaldson & Shapiro, 2010). In our study, the work pressure may also have led to nurses leaving out time‐consuming steps such as scanning patients or medications (van Onzenoort et al., 2008).

Workarounds were associated with the time of the medication round and particular days. Workarounds seem to be more likely on busy weekdays versus the relatively quiet Sunday. Also, workarounds were more likely on the rounds scheduled during the afternoon and evening. This may also be due to the busier parts of the day, leading to nurse workarounds to save time. Our findings emphasise the need to review the patient–nurse ratio, work schedules and medication‐related workload per day of the week and per shift to ensure the safe use of the system. Nurse managers are responsible for a positive work environment and for planning an adequate balance between patients and available nursing care (van Oostveen, Braaksma, & Vermeulen, 2014).

Interestingly, we found several factors such as work experience of nurses and the percentage of barcoded medications not associated with workarounds. Work experience of nurses has been found to be associated with the quality of performance (Blegen, Vaughn, & Goode, 2001) but obviously did not play a role in how nurses dealt with recently introduced IT‐based systems as barcode administration. Efforts to prevent workarounds should be therefore targeted to all nursing staff involved, independent of their work experience. Another reason could be found in our dichotomous analysis of the nurse experience. A further refinement of the work experience would possibly have shed light on this item.

Our finding that the percentage of barcoded medication was a factor not associated with workarounds is noteworthy. In the Netherlands, about 80% of the medication in hospitals is barcoded by the vendor (single‐cell medication). Especially, liquids, eye drops and eardrops and ointments are not provided with single‐cell barcodes. For reasons of patient safety, a lot of effort is taken by hospitals to apply their barcodes to individual medication without those vendor‐supplied barcodes. Since the percentage of barcodes was not associated as a factor in carrying out workarounds by nurses, current efforts seem to be sufficient in this respect.

### 6.1 Strengths and limitations

A strength of our study is that we quantitatively assessed the association of potential risk factors with workarounds in a large sample from multiple institutions using a robust method of data collection. The multicentre design of the study enhances the generalisability of our data.

Our study has some limitations, as well. Despite disguised observation being considered as the “golden standard” of data collection in medication administration error studies (Barker, Flynn, & Pepper, 2002; Dean & Barber, 2001; Westbrook & Ampt, 2009), observation bias cannot be excluded: observers may become tired and therefore less precise. We educated the observers intensively and trained them to stay nearby to the nurses managing the medication. Only a limited quantity (228, 3.8%) of observations had to be excluded due to the incompleteness of the observation. Furthermore, the observer may have influenced the nurses, but this phenomenon known as “Hawthorne effect” is reported to be small (Gale, 2004). Also, observers may have missed some workarounds. An ethical question that can be raised in our research is whether observers have the right to observe persons who are not fully aware of the nature of the data that are collected. However, in addition to the national permission for this study, every participating hospital was informed (both the board of directors and ward management) as well and received copies of the research protocol and the nationwide approval of our research, and no single objection was noted. Other limitations were that all four hospitals had BCMA software systems from different vendors (van der Veen, van den Bemt, Bijlsma, et al., 2017), and we observed only nurses from internal medicine and surgical wards and patients aged 18 years and older. Finally, we based the selection of potential risk factors on literature. Hence, we cannot rule out that we have missed some factors. Exploring nurses' beliefs and attitudes using BCMA may reveal additional user aspects, as has been shown in a study on double‐checking procedures (Schwappach, Pfeiffer, & Taxis, 2016). Using the Australian Work Observation Method by Activity Timing (WOMBAT) technique (Westbrook, 2009; Westbrook & Ampt, 2009) may be another way to gain a better understanding of the underlying causes of some of the factors (Westbrook, Duffield, Li, & Creswick, 2011).

### 6.2 Further research

Our results suggest that workload may be an important cause of workarounds. One example of a workload reducing intervention could be the introduction of dedicated personnel—such as pharmacy technicians—who are solely responsible for medication administration. Pharmacy technicians are trained to handle medication as the main part of their daily work, in contrast to nurses for whom medication administration is only a part of their daily routine. In addition to this, pharmacy technicians, given the nature of the work in the pharmacy, might be better trained in the use of technology in general. Research from both the USA and the UK shows the feasibility of medication administration to hospitalised patients by pharmacy technicians (Keers, 2017; Pedersen, Schneider, & Scheckelhoff, 2012).

On the other hand, this could be costly, and pharmacy technicians would have less of a sense of the patient and their conditions. Cluster randomised controlled trials (cluster RCTs) (Harris et al., 2018) or interrupted time series (Westbrook, Raban, Lehnbom, & Li, 2016) are needed to demonstrate the effectiveness of this intervention. Further research should also include qualitative research methods, for example interviewing nurses responsible for medication administration using IT‐based interventions as BCMA, exploring further the causes for workarounds.

## 7 CONCLUSIONS

Nurses administering medication using BCMA frequently performed workarounds. Potential risk factors associated with these workarounds were the administration of nonoral drugs, medication from ATC classes that were infrequently given, the day of the week, the time of the medication round and the patient–nurse ratio. Especially, the nurse workload reflected in the day of the week, time of the medication round and patient–nurse ratio could be the focus for improvement measures as these are the most clearly modifiable factors identified in this study.

## 8 RELEVANCE TO CLINICAL PRACTICE

Our study has identified several potential risk factors of workarounds, which may compromise medication safety. Especially, the patient–nurse ratio and certain—potential busier—moments of the day and week were associated with performing nurse workarounds. These factors could be the focus for improvement measures aimed at reducing the workload per nurse.

## AUTHOR CONTRIBUTION

Willem van der Veen, Patricia M.L.A. van den Bemt and Katja Taxis each contributed equally to the design, the analysis and the interpretation of the data of the study, drafted the manuscript and agreed to be accountable for all aspects of the work in ensuring that questions related to the accuracy or integrity of any part of the work are appropriately investigated and resolved. Hans Wouters, Hester Vermeulen and David W. Bates made each equally substantial contributions to the analysis and interpretation of the data of the study and the drafting of the final manuscript. All authors read and approved the final manuscript and agreed to be accountable for all aspects of the work in ensuring that questions related to the accuracy or integrity of any part of the work are appropriately investigated and resolved.

## ETHICAL APPROVAL

The study is approved by the “Regionale Toetsingscommissie Patientgebonden Onderzoek RTPO” (22 May 2014, reference number RTPO 920a). The study was registered in the “Dutch trial register” with trial ID NTR4355.

## APPENDIX 1

### BCMA Study Group

Michiel Duyvendak, PharmD, PhD, Hospital Pharmacist, Department of Hospital Pharmacy, Antonius Hospital, Sneek, the Netherlands. Karen Oude Luttikhuis, MSc, PharmD, Goorse Apotheek, Goor, the Netherlands. Johannes J. W. Ros, MSc, PharmD, Hospital Pharmacist, Department of Clinical Pharmacy, Gelre Hospitals, Apeldoorn, the Netherlands. Erwin C. Vasbinder, PharmD, Ph.D., Hospital Pharmacist, Department of Hospital Pharmacy, Groene Hart Hospital, Gouda, the Netherlands. Maryam Atrafi, MSc, Utrecht University, school of Pharmacy, Utrecht, the Netherlands. Bjorn Brasse, MSc, PharmD, Hospital Pharmacist, Department of Hospital Pharmacy, Elkerliek Hospital, Helmond, the Netherlands. Iris Mangelaars, MSc, PharmD, Utrecht University, School of Pharmacy, Utrecht, the Netherlands.
